# Report of the Proceedings of the Pennsylvania Society of Dental Surgeons

**Published:** 1848-10

**Authors:** 


### ARTICLE IX.

### *Report of the Proceedings of the Pennsylvania Society of Dental Surgeons.*

The Society met at 7½ o'clock, at the Hall of Pharmacy, Oct. 3, 1848. President, Dr. E. Parry, in the Chair, and Mr. A. R. Johnson, Secretary.

Minutes previous meeting read and adopted.

Treasurer's report now read, and a Committee appointed to audit, who reported a very favorable state of the finances.

Chairman of the Examining Committee handed in a committee report in favor of receiving Mr. W. R. White into membership.

The Committee to whom was referred *Lawrence's Tongue Holder*, reported through their Chairman, Dr. J. D. White, as follows:

### *Report of Committee on Lawrence's Tongue Holder.*

*To the President and Members of the*
*Pennsylvania Association of Dental Surgeons.*

Gentlemen—Your Committee appointed to investigate the merits of Lawrence's Tongue Holder, respectfully beg leave to announce that they have attended to that duty. The Committee, therefore, respectfully report, that as far as they have tried it, they find it to be very useful in many prolonged operations in plugging the teeth, as an assistant in keeping away the tongue and saliva; but on account of its depressing the tongue too much, in many cases, and forcing it against the fauces, and producing nausea, it is not as useful in general practice as was at first anticipated by your Committee; still there are many instances in which it will be found to be almost indispensable. While every compliment is due to Mr. Lawrence for the very liberal manner in which he has laid it before your Committee, yet they would be doing injustice to a highly respectable French author, M. Desirabode, (who has used and described the same contrivance precisely, as will be observed by a reference to page 262 of the American Library of Dental Science, for 1847,) if they were to regard the principle of Mr. Lawrence's apparatus as anything more than identical with M. Desirabode's. However, the Committee do not wish to be understood as believing that Mr. Lawrence did not invent it without the knowledge of its former existence. The following is the description of the instrument, as given by M. Desirabode: "The tongue may be kept out of the way by means of a fixture, made of two semi-elliptical plates of boxwood, ivory or platina, applied, one to the roof of the mouth, the other placed upon the tongue, and kept apart by a piece of whalebone, curved backwards and fixed by each end into sheathes made in the plates to receive them.

The superior plate is terminated by an appendage which touches the posterior border of the alveoli and the teeth in order to make a projection out of the mouth, by which the apparatus may be removed with facility and promptitude in cases where it forms an obstacle which cannot be immediately removed by the fingers."

*Resolved*, That a vote of thanks be awarded to Mr. Lawrence for presenting the subject to the consideration of the Society.

All of which is respectfully submitted.

Signed, J. D. White, M. D.
S. T. Beale, M. D.
Mr. C. C. Williams.

The resolution was adopted.

Next in order was the report of the Committee on *Gilbert's Central Cavity Plate :*

***To the President and Members of the***
***Pennsylvania Association of Dental Surgeons :***

Gentlemen—The Committee appointed by you to examine into the merits of "Gilbert's Patent Cavity Plate," respectfully beg leave to report that they have attended to the duties assigned them as far as in their judgment is necessary. With reference to the priority of invention of this plate, your Committee do not pretend definitely to report, inasmuch as numbers claim the originality from ten to fifteen years back; still there does not seem to be any evidence of it, except their own assertions. However, some operators have constructed a plate with a number of chambers, and consider it to have been done for the same purpose as the single chamber claimed by Gilbert, or in other words, that the invention of one is equivalent to the invention of the other, and that substituting one chamber for any number, does not entitle the modification to the credit of originality. Now inasmuch as Mr. Gilbert was the first (as far as your Committe are aware) to make the Cavity Plate public, he is entitled to the credit of the invention, so far as it subserves the public good, for we make no doubt that those who have been capable of confining it to the secrets of their own closets for fifteen years, would do so that much longer; however, your committee will leave that part of the subject, believing that his patent papers will protect him against any unjust attacks from pretending claimants.

With regard to its practical uses, your committee would report, that in a great number of cases, it has been most markedly successful, and in cases, too, where springs had been unsuccessfully applied by different operators, and they believe also that this happy result has been from the use of the "Central Cavity Plate."

This central cavity seems to be a kind of "neutral ground" or reservoir, as well for atmospheric air as the elasticity of the gum, and it is well known that the alveolar process is constantly undergoing slight absorption until it entirely disappears, and, that when the plate extends over the entire surface of the hard palate, it will sooner or later impinge with more force upon it, than upon the alveolar ridge, and produce a rocking motion of the artificial teeth, a difficulty which this central cavity in a measure prevents, and as there is some elasticity in the gum at all times, unequal pressure upon any part of the operation will produce a rocking motion, even of a well-fitted plain plate, or a plate with cavities upon opposite sides; the hard palate will act as a pivot upon the central part of a plain plate, from the yielding character of the gums over the alveolar process, and destroy the full influence of the atmospheric pressure in many cases. This cavity plate can be applied in a large number of cases for setting a single tooth, or an indefinite number, as the accompanying specimen will illustrate.* It is an instance where two teeth have been injured by clasps, but which are now worn with entire comfort and usefulness. By an examination of a great number of cases which have been worn from four to seven months, and kept in the mouth constantly, (except while cleansing them,) no irritation of the hard palate was observable, or unpleasant consequences in any respect. In some few cases where it has not been entirely satisfactory, it seems to have resulted rather from an improper adaptation of the plate, condition

*We saw the plate above referred to.—*Ed.*

of the mouth, or an inability of the patients to accommodate themselves to it, than a want of power in the plate; notwithstanding it is believed where springs have been applied, the cases are worn with greater usefulness than had the cavity not been used also.

*Resolved*, That a certificate of approval of the Central Cavity Plate, should be awarded to Mr. Gilbert, by this Society.

This was approved, and received the signatures of the officers of this Society.

Signed, J. D. White, M. D.<br>

S. T. Beale, M. D. } Committee.<br>

Ely Parry, M. D.

On motion of Dr. J. D. White, a committee was appointed to report a plan for the establishment of a Cabinet and Library, for the Society. Drs. E. Parry, J. D. White, C. C. Williams, F. Reinstein and A. R. Johnson, the committee, with power to add.

An interesting debate here sprung up on the action, where two metals are used in one filling, such as gold and tin, the saliva acting as a menstruum or medium, and where the baser metal is oxydized by exhalents and by imbibition through the bony structure of the tooth. Many facts were brought out, and much information obtained.

In conclusion, we may add, the right spirit is manifested by the members, and every thing bids fair to place the Society in that position, whence much good may be disseminated among the members.—*Dental News Letter.*

